# Genome Sequencing and Metabolic Potential Analysis of *Irpex lacteus*

**DOI:** 10.3390/jof10120846

**Published:** 2024-12-07

**Authors:** Yue Wang, Yingce Duan, Menghan Zhang, Chaoqin Liang, Wenli Li, Chengwei Liu, Ying Ye

**Affiliations:** 1 Key Laboratory for Enzyme and Enzyme-Like Material Engineering of Heilongjiang, College of Life Science, Northeast Forestry University, Harbin 150040, China; wangyue20000920@163.com (Y.W.); yingceduan@163.com (Y.D.); z15200085936@163.com (M.Z.); 2 Hubei Key Laboratory of Natural Medicinal Chemistry and Resource Evaluation, School of Pharmacy, Tongji Medical College, Huazhong University of Science and Technology, Wuhan 430030, China; d202482102@hust.edu.cn; 3 State Key Laboratory for Crop Stress Resistance and High Efficiency Production, Shaanxi Key Laboratory of Natural Products & Chemical Biology, College of Chemistry & Pharmacy, Northwest A&F University, Yangling 712100, China; liwenli@nwafu.edu.cn

**Keywords:** *Irpex lacteus*, genome sequencing, CAZymes, P450, secondary metabolites

## Abstract

*Irpex lacteus* is an edible and medicinal macrofungus with significant biological activity and broad pharmaceutical prospects that has received increasing attention in recent years. Although it is an important resource for macrofungi, knowledge of it remains limited. In this study, we sequenced, de novo assembled, and annotated the whole genome of *I. lacteus* using a PacBio Sequel II sequencer. The assembled 41.83 Mb genome contains 13,135 predicted protein-coding genes, 83.44% of which have searchable sequence similarity to other genes available in public databases. Using genome-based bioinformatics analysis, we identified 556 enzymes involved in carbohydrate metabolism and 103 cytochrome P450 proteins. Genome annotation revealed genes for key enzymes responsible for the biosynthesis of secondary metabolites, such as terpenoids and polyketides. Among them, we identified 14 terpene synthases, 8 NRPS-like enzymes, and 4 polyketide synthases (PKS), as well as 2 clusters of biosynthetic genes presumably related to terpene synthesis in *I. lacteus*. Gene family analysis revealed that the MYB transcription factor gene family plays an important role in the growth and development of *I. lacteus*. This study further enriches the genomic content of *I. lacteus*, provides genomic information for further research on the molecular mechanism of *I. lacteus*, and promotes the development of *I. lacteus* in the fields of drug research and functional food production.

## 1. Introduction

*Irpex lacteus*, belonging to the phylum Basidiomycota, class Agaricomycetes, order Polyporales, family Irpicaceae, genus Irpex, is a medicinal fungus [1]. *I. lacteus* is also a common wood-rotting fungus, and its substrate grows mainly on dead standing trees and broad-leaved fallen wood; it is often flat-volumed and recoiling, occasionally lateral. The cap is often imbricated, shell-shaped, or united with the side. The upper surface is white to cream-colored or yellowish, with dense downy hairs, and has an inconspicuous ring band, smooth with shallow grooves, with an edge of the same color; the aperture surface is white to cream-colored and angular. The flesh of the fungus is white to pale, soft and fibrous, without annular bands, and it has a distinctive light fragrance that disappears when dried [2]. *I. lacteus* is distributed in Jilin, Heilongjiang, Hebei, Jiangxi, Shanxi, Sichuan, Yunnan, Tibet, and other places in China [3]. In recent years, researchers have isolated and obtained various types of secondary metabolites in *I. lacteus*, such as terpenoids [4], polyketides, and furan derivatives [5], which have a variety of physiological activities, such as anti-inflammatory, inhibition of lymphocyte proliferation, and NO production. For example, Tremutin A inhibits the lipopolysaccharide (LPS)-induced proliferation of B lymphocyte cells. Tremutin B inhibits concanavalin A (Con A)-induced T cell proliferation and LPS-induced B lymphocyte cell proliferation [6]. Irpeksin A-E showed significant inhibitory activity against NO production in LPS-activated RAW 264.7 macrophage cells with IC50 values varying from 2.2 to 19.6 Μm [7]. Irpexlacte A-D showed moderate antibacterial activity against *Pseudomonas aeruginosa*. Irpexlacte A and D showed remarkable antioxidant activity [8]. As a medicinal fungus, the crude polysaccharide fraction of *I. lacteus* was approved by the China Food and Drug Administration and named YiShenKang, which is used clinically for the treatment of chronic glomerulonephritis [9]. All these data indicate that *I. lacteus* is a fungus with great potential for use. In addition to pharmaceutical uses, it has good application prospects in industry, agriculture, and environmental pollution control. Some studies have reported that *I. lacteus* shows good degradation of garden tree branches [10], a good decolorization and detoxification effect in the treatment of wastewater containing pigments [11], and it can also biologically pre-treat corn stover [12]; thus, it is a white-rot fungus suitable for water and soil bioremediation. Due to its white-rot fungal properties, it is currently considered as the most important and promising lignocellulose degrading organism [13]. The fermentation broth of *I. lacteus* during the fermentation process gives off an aroma, and more than 30 spice components can be detected using analytical techniques such as GC-MS, which are used in the field of daily chemicals and food products [14]. Despite the growing interest in the active constituents of *I. lacteus*, little is known to date about the molecular and genetic mechanisms of the biosynthetic pathways that produce these constituents due to the limitations of genome assembly.

Genomics research provides a window into understanding species. Recently, third-generation sequencing technologies have emerged that offer higher throughput, shorter sequencing times, longer read lengths, and lower costs, allowing researchers to explore genomes with unprecedented resolution and permitting more accurate and comprehensive genome sequencing of medicinal and edible fungi [15]. This has facilitated human research on various aspects of these fungi, including their life cycles, nutritional patterns, mating types, and biosynthetic pathways of bioactive metabolites. As a result, the genomes of some valuable fungi including *Hericium erinaceus* [16], *Inonotus obliquus* [17], *Agaricus bitorquis* [18], *Ganoderma lucidum* [19], and *Laetiporus sulphureus* [20] have been successfully analyzed, which further helps to develop their medicinal value and promote industrial development. To understand the genetic factors of *I. lacteus*, provide genomic data for further study of its biological functions, and promote the mining of medicinal active ingredients and industrial development of *I. lacteus* from a genomic perspective, we used the PacBio Sequel II sequencer to perform a de novo genome-wide sequence analysis of *I. lacteus* and perform a high-quality assembly of its genome. The high-quality genome sequence allowed us to identify functional genes involved in secondary metabolite biosynthesis, deepening our understanding of the mechanisms of secondary metabolite biosynthesis, and providing some new insights into growth, development, and carbohydrate degradation in this species.

## 2. Materials and Methods

### 2.1. Collection of Strains and Culture Conditions

The fruit body was collected from the Changbaishan Forest District, Jilin Province, on rotting wood and named *Irpex lacteus* Y1, which was identified based on internal transcribed spacer sequences (ITS1 and ITS4) after tissue separation (Figure S1). The strain was cultured in potato dextrose broth at 25 °C for 7 days. Slant genomic DNA was extracted from mycelium using the Tiangen plant DNA kit DP350(Beijing Tiangen Biochemical Technology Co., Beijing, China), according to the manufacturer’s instructions.

### 2.2. Genome Sequencing and Assembly

Genomic DNA was sequenced using the PacBio Sequel II sequencing platform (Pacific Biosciences, CA, USA). The software SMRTlink8.0 (https://www.pacb.com/support/software-downloads/, accessed on 21 June 2022) was used to filter and process the downstream data, while the parameter --minLength=50 was set to filter out sequences below 50 bp to obtain consensus reads. Genome sequence splicing of Hifi sequencing reads was performed using Hifiasm (v1.6) software [21]. Polish correction was performed by matching the three-generation data to the assembly results using Racon (v1.4.20) (https://github.com/lbcb-sci/racon, accessed on 21 June 2022) software, and the final two-generation data were matched to the corrected genome sequence using Pilon (v1.22) (https://github.com/broadinstitute/pilon, accessed on 21 June 2022) to obtain the final corrected sequence. The corrected sequences were evaluated for assembly results using Quast (v5.1.0rc1) (http://quast.sourceforge.net/quast, accessed on 21 June 2022) software.

### 2.3. Gene Prediction and Annotation

Gene prediction of fungi was performed using Genemark_ES (v4.46) (http://exon.gatech.edu/GeneMark/, accessed on 22 June 2022) software [22], which mainly includes gene number, total gene length, GC content, gene percentage of genome, average gene length, intergenic region length, intergenic region GC content, and intergenic region percentage of genome. The results were analyzed by RepeatMasker. Repeat sequence prediction of fungal genome was performed using RepeatMasker (v4.0.7) (http://www.repeatmasker.org/, accessed on 22 June 2022) software [23]. Barrnap (v0.4.2) (https://github.com/tseemann/barrnap, accessed on 22 June 2022) and TRNAscan-SE (v1.3.1) (https://lowelab.ucsc.edu/tRNAscan-SE/, accessed on 22 June 2022) software were utilized [24], respectively, for the fungal genomes containing the rRNA and tRNA that were predicted. The protein sequences of the predicted genes were Blastp (BLAST 2.2.28+) compared with functional databases such as GO [25], KEGG [26], COG/KOG [27], and NR [28], respectively, to obtain the annotation information of the predicted genes.

### 2.4. CAZy and CYP Family in I. lacteus

The CAZy [29] database collects various carbohydrate enzymes containing six families of glycoside hydrolases (GHs), glycosidyltransferases (GTs), polysaccharide lyases (PLs), glycohydrolase esterases (CEs), carbohydrate-binding modules (CBMs), and auxiliary module enzymes (AAs). The structural information of various enzyme classes contained in the CAZy database was identified in the obtained protein sequences using the HMMER (version:3.2.1, filter parameter E-value < e^−5^ coverage > 0.35) method to characterize the enzyme classes contained in the *I. lacteus.* Carbohydrate-related enzyme genes were identified in *I. lacteus*, and these were categorized into six major groups according to species.

The gene set was compared with the CYP450 (https://p450.riceblast.snu.ac.kr/intro.php, accessed on 24 June 2024)) database using BLASTP, and the comparison parameter was set with an expectation E-value of 1 × 10^−5^ to obtain the gene corresponding CYP450 functional annotation information. The screened CYP450 was subjected to structural domain prediction in NCBI Conserved Domain, and proteins with CYP450 transmembrane structural domains were screened. Three similar species, *Coprinopsis cinerea*, *A. bisporus*, and *Pleurotus ostreatus*, were selected from the fungal P450 database, and the P450 gene sequences were chosen as references for comparison and clustering of P450 from *I. lacteus*. Phylogenetic tree analysis of 106 more numerous and well-classified P450s was performed using the same method.

### 2.5. Prediction of Gene Clusters Involved in Secondary Metabolites

The network analysis platform antiSMASH6.0 (http://antiSMASH.secondarymetabolites.org/, accessed on 24 June 2024) [30] and the network analysis tool 2ndFind (http://biosyn.nih.go.jp/2ndFind, accessed on 23 June 2024) were utilized to predict secondary metabolism biosynthesis genes as well as gene clusters. Both used default parameter settings. To validate the prediction results, blastp analysis and gene annotation were performed using the NCBI platform. We searched all hypothesized gene models in the database using the blastp and tblastn algorithms.

### 2.6. Bioinformatics and Phylogenetic Analyses of STSs, PKSs, and P450s

Nineteen sesquiterpenes from *Omphalotus olearius*, *C. cinereus*, and *Stereum hirsutum* were selected to classify twelve STSs. A maximum likelihood tree comparing 31 sequences was built using MEGA with 1000 bootstrap settings. Homologous PKS sequences of 33 different fungal species were retrieved from the NCBI and JGI databases, and most of these sequences had completed functional validation. Sequences of the KS structural domains of the PKSs were aligned using Clustal X (Version 2.0) and maximum likelihood trees were generated using MEGA (Version 10.0) software. Structural domain features were analyzed using Synthaser [31].

### 2.7. MYB TFs Gene Family Analysis

Based on the whole genome data of *I. lacteus*, the MYB structural domain (PF00249) was used as a search model by using the Pfam (http://pfam.xfam.org/search, accessed on 24 July 2024) tool, and the HMM 3.0 software was used to screen for MYB transcription factors in Poria cocos that contain this structural domain. Online software such as Conserved Domain Database (CDD) and SMART 8.0 (http://smart.embl-heidelberg.de/, accessed on 24 July 2024) from the NCBI database were further utilized to screen and confirm the presence of the SANT structural domain in the ILMYB protein sequence. The conserved motif of the ILMYB protein was predicted using the online tool MEME (http://meme-suite.org/index.html, accessed on 25 July 2024), with the number of motif lookups set to 10 and all other parameters set to default values. The promoter sequence of the ILMYB gene 2000 bp upstream of the ATG was extracted and the cis-acting elements of the promoter region were analyzed using the Plant CARE online website (http://bioinformatics.psb.ugent.be/webtools/plantcare/html/, accessed on 25 July 2024). The 14 ILMYB protein sequences were used to construct an evolutionary tree with MYB sequences of *G. lucidum*, *P. ostreatus*, *Cordyceps sinensis*, and *Aspergillus fumigatus* using the neighbor-joining (NJ) method using MEGA 7.0 software, with the bootstrap method set to 1000 and the rest of the parameters set to the default values.

## 3. Results

### 3.1. Genome Sequence Assembly and Annotation

**Figure 1:**
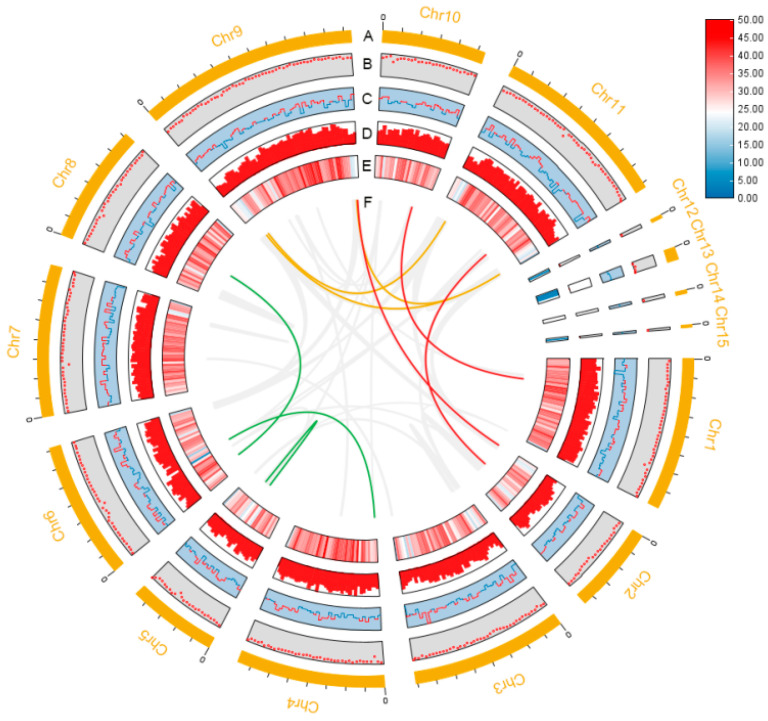
Genome diagram of *I. lacteus* genome. (**A**) Chromosome length; (**B**) GC ratio; (**C**) GC skew; (**D**) and (**E**) gene density; (**F**) collinearity analysis.

The genome assembly produced a total of 3,416,337 subreads, with an average subreads length of 11,877 bp; these reads assembled into a high-quality genome. The genome size was 41.83 Mb, consisting of 55 contigs, an N50 of 3.95 Mb, and a GC content of 49.82% (Figure 1, Table 1). Quast (v5.1.0rc1) evaluation showed the largest contig length of 5,693,878 with a mismatch of 0. According to Figure S2, the scatterplot showed a shape approximating a Poisson distribution, indicating that the GC bias was not severe during the sequencing process. These results indicate that the genome assembly is of good quality. A total of 13,135 protein-coding genes were predicted from the *I. lacteus* genome, with an average CDS sequence length of 1530 bp and a total linkage length of 201,052,286 bp, which accounted for 48.06% of the whole genome (Table 2). For non-coding RNAs, 364 tRNAs and 77 rRNAs were predicted. Based on homology comparison, a total length of 452077 bp of repetitive sequences was identified using RepeatMasker (v4.0.7), which accounted for 1.08% of the *I. lacteus* genome. Also identified were 62 small RNAs, accounting for 0.25% of the whole genome; 5760 simple repeats, accounting for 0.66% of the whole genome; and 1366 low complexity, accounting for 0.17% of the whole genome.

To obtain more comprehensive information about gene functions and achieve a comprehensive functional annotation of protein-coding genes, similarity analysis was performed on 13,135 non-redundant genes based on five public databases: NR, COG, KOG, KEGG, and GO, to reveal their functional diversity across these databases. The annotation results of the Nr library showed that a total of 10,960 genes were annotated in more than 20 species (Figure S3), accounting for 83.44% of the total number of protein-coding genes, with the Kyoto Encyclopedia of Genes and Genomes (KEGG; 7639 genes/58.16%), Gene ontology (GO; 5627 genes/42.84%), and Clusters of Orthologous Groups (COG; 3974 genes/30.26%).

Based on the COG database (Figure S4), a total of 6537 genes were identified, of which 541 belonged to group R, the most prominent group related to general function prediction. The second group, “Replication, recombination and repair” was associated with the majority of genes. “Carbohydrate transport and metabolism”, “Posttranslational modification, protein turnover, chaperones”, and “Secondary metabolites biosynthesis, transport and catabolism” are the most gene-rich categories in the COG group. These results suggest that there is abundant metabolism of proteins, lipids, and sugars, resulting in higher energy conversion efficiencies, and that these relatively abundant energy pathways can promote growth and reproduction in *I. lacteus*.

The annotations in the GO database allow us to gain insight into the biological significance represented by the genes. GO functional analysis shows (Figure S5) that the annotated genes are distributed across three functional categories: biological process, cellular component, and molecular function. The categories include “cellular process” (5080); “metabolic process” (4723) and “biological regulation” (3625) from biological processes; “cell” (5210), “cell part” (5210), and “organelle” (4829) from cellular components; and “binding” (4231), “catalytic activity” (3694), and “transporter activity” (632) from molecular function.

The annotation of the KEGG pathway database can systematically analyze the functions of gene products and the metabolic pathways of these products in cells. It helps us to understand the biological functions of genes and complex biological processes at a systematic level. The KEGG database identified 7639 genes involved in six pathways, among which the number of genes involved in metabolic pathways is the highest (Figure S6).

### 3.2. CAZymes Analysis

**Figure 2:**
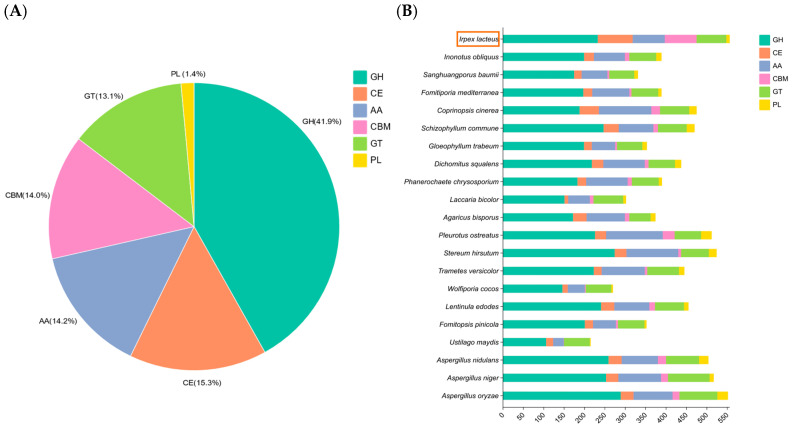
CAZymes analysis of *I. lacteus*. (**A**) Gene distribution of *I. lacteus* based on the six major modules of CAZymes; (**B**) results of CAZyme profiling of 21 fungal species.

CAZymes are one of the most important gene families in the fungal genome and play a crucial role in fungi, where they are responsible for the degradation of lignocellulose, as well as many other biological processes, such as development and stress responses [32,33]. CAZymes in basidiomycetes help degrade lignin and polysaccharides (cellulose and hemicellulose) in the biomass to obtain the carbon sources needed for their life activities [34,35]. In the present study, 556 genes encoding sugar-activating enzymes (CAZymes) were found in the *I. lacteus* genome (Figure 2A), including 233 glycoside hydrolases (GHs), 85 carbohydrate esterases (CEs), 79 auxiliary activities (AAs), 78 carbohydrate-binding modules (CBMs), 73 glycosyltransferases (GTs), and 8 polysaccharide lyases (PLs). GHs are primary enzymes that cleave the glycosidic bonds in cellulose and hemicellulose, while AAs usually act synergistically with GHs. Most of the identified proteins with structural domains of the GH5, GH6, GH7, GH8, GH9, and GH12 families can act on cellulose; the GH18 and GH19 families act on chitin, and the nine AA1-3, AA5-AA9, and AA14 families act on cellulose and hemicellulose [36]. The GHs-like proteins of *I. lacteus* were mainly distributed in GH5, 16, and 18, while the AAs were mainly distributed in AA3, 7, and 9, resulting in its high lignocellulose degradation capacity. Additionally, we analyzed the CAZyme profiles of 21 fungal species. We found that *I. lacteus* had more carbohydrate genes than white-rot fungi, such as *Phanerochaete chrysosporium*, and umbrella-type wood-rot fungi such as *P. ostreatus* and *Lentinula Edodes* (Figure 2B).

### 3.3. Mining of Biosynthetic Genes of Secondary Metabolic

**Figure 3:**
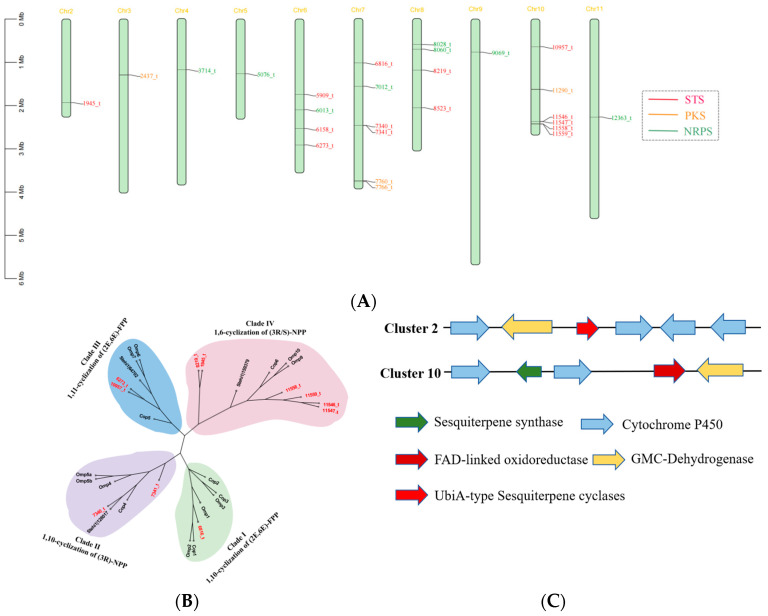
Analysis of genes involved in secondary metabolite biosynthesis. (**A**) Distribution of biosynthetic core genes for natural products on the chromosomes; (**B**) phylogenetic analysis of sesquiterpene synthase (STS) homologues; (**C**) schematic diagram of the composition of postulated clusters 2 and 10.

Given the significant medicinal value of *I. lacteus*, we analyzed the biosynthetic potential of its secondary metabolites. Genome prediction of strain Y1 using the web tool antiSMASH revealed that the genome contained 26 core genes and 2 secondary metabolite biosynthesis gene clusters (BGCs). The core genes consisted of 14 terpene synthases, 8 NRPS-like enzymes, and 4 polyketide synthases (PKSs) distributed across 10 chromosomes (Figure 3A, Table 3).

#### 3.3.1. Terpenoid Biosynthesis

**Figure 4:**
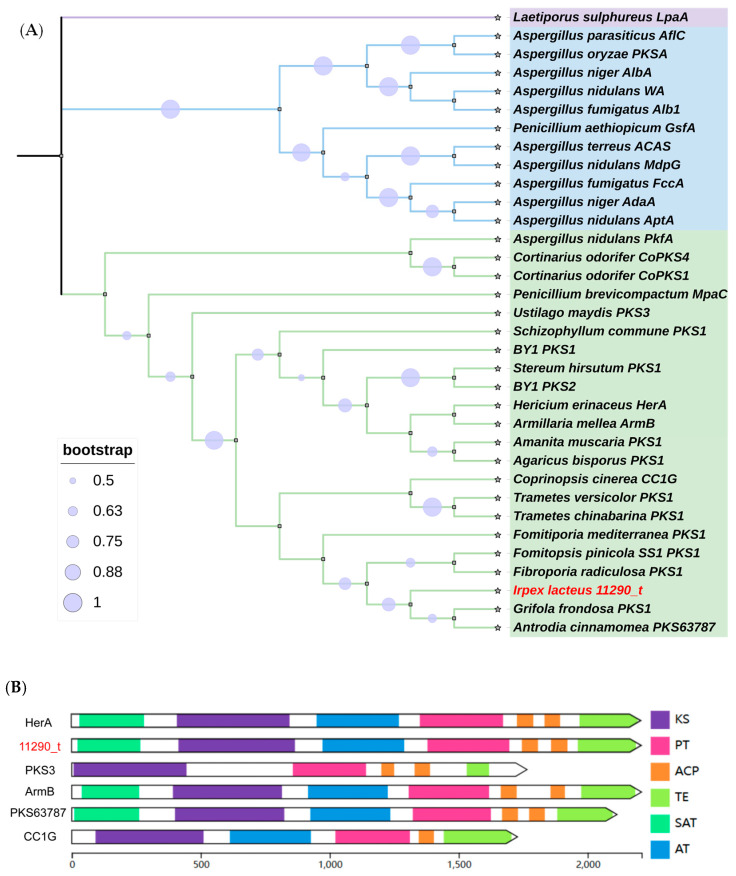
Analysis of the gene for polyketide synthase from *I. lacteus*. (**A**) Phylogenetic tree of different functional PKS enzymes constructed by maximum likelihood analysis of the keto-synthase (KS) domain amino acid sequences; (**B**) structural domains of orsellinic acid synthase from several species of basidiomycete.

Terpenes are one of the major secondary metabolites of *I. lacteus*, accounting for more than two-thirds of the reported secondary metabolites of *I. lacteus*. In this study, we identified 14 terpene synthase-related genes in the *I. lacteus* genome, including 12 sesquiterpene synthases, 1 lanosterol synthase, and 1 squalene synthase. Based on the same conserved structural domains, we identified 12 genes potentially involved in sesquiterpene synthases. We used other known sesquiterpene synthases such as *O. olearius* [37] and *S. hirsutum* [38] as identification criteria for the type of sesquiterpene synthase in *I. lacteus*. The 12 sesquiterpene synthases can be divided into four clades (Figure 3B). Six sesquiterpene synthases belonged to Clade IV, two belonged to Clade II, two belonged to Clade III, and one sesquiterpene synthase belonged to Clade I. The enzymes were classified into four clades (Figure 4B). The STS in Clade I can utilize (2E,6E)-FPP as a substrate to produce sesquiterpenes derived from the E, E-germacradienyl cation via a 1,10 cyclization reaction. Clade II consists of enzymes that share the cyclization mechanism of 1,10-cyclization of (3R)-nerolidyl diphosphate, producing a 10-membered cyclic sesquiterpene derived from the Z, E-germacradienyl cation. Clade III consists of enzymes believed to share a common 1,11-cyclization mechanism of (2E,6E)-FPP, producing the trans-humulol cation. Clade IV consists of enzymes believed to share a common 1,6-cyclization mechanism of (3R/S)-NPP, producing sesquiterpenes derived from (6R)-β-bisabolol cation [39]. Cluster 2 contains a sesquiterpene synthase gene, two P450s, and two oxidoreductase genes, which suggests that Cluster 2 may encode structurally complex products. Cluster 10 contains a terpene cyclase of the UbiA class, four P450s, and one GMC; the multiple post-P450-modifying enzymes may yield structurally diverse secondary metabolites (Figure 3C).

#### 3.3.2. Polyketide Biosynthesis

**Figure 5:**
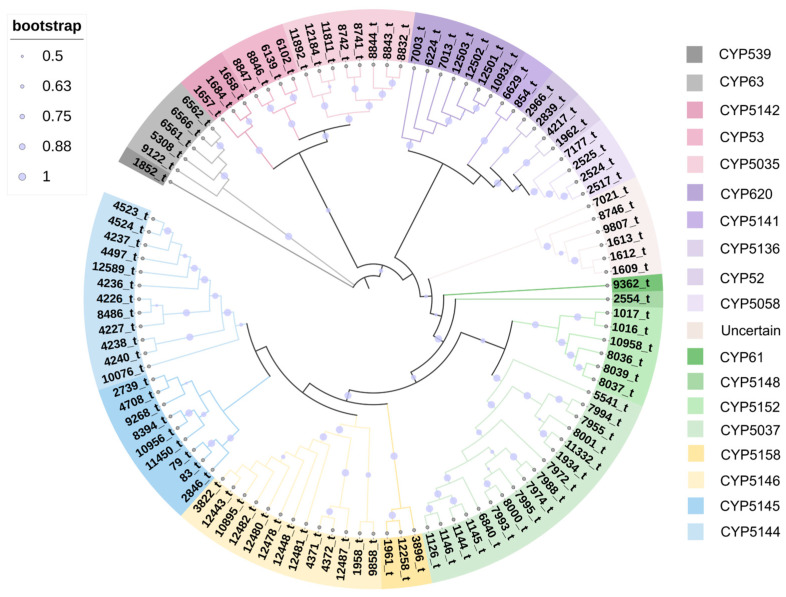
Maximum likelihood tree of 106 cytochrome P450s from *I. lacteus*. Each cytochrome P450 family is shown in a separate color, and the branch reliability value of over 50 is marked on the corresponding branch node.

Polyketides are a class of structurally diverse secondary metabolites produced by bacteria, fungi, plants, and animals. They include medically important antibiotics (e.g., tetracycline, erythromycin), antitumor agents (e.g., epothilone), and the lipid-lowering drug lovastatin [40,41]. To date, three types of PKS have been identified, with iterative type I PKS predominating in fungi. We found that the number of PKS in basidiomycetes fungi is low and much less than that in Ascomycetes, and their products are relatively homogeneous and structurally less rich than those produced by Ascomycetes. So far, fewer studies have been conducted on PKS in basidiomycetes, mainly *C. cinerea* PKS (CC1G_05377) [42], *Armillaria mellea* ArmB [43], *Antrodia cinnamomea* PKS63787 [44], and *H. erinaceus* HerA [45], which are responsible for the biosynthesis of orsellinic acid. *Ustilago maydis* PKS5 [46] converts orsellinic acid to orsellic aldehyde. Orsellinic acid (OA) is a dihydroxybenzoic acid derivative with an extra methyl group, and its natural derivatives have important biological activities, such as antroquinonol from *Antrodia camphorata*, which has non-small cell tumor inhibitory activity and is currently a phase II clinical lead drug [47]; *Acremonium egyptiacum* produces ascofuranone (AF), a promising drug candidate for the treatment of African trypanosomiasis and a potential anticancer lead compound [48]. We retrieved four PKS genes from the genome of *I. lacteus*, and by sequence comparison, we found that one PKS, *11290_t*, shares 60% sequence similarity with *armB*, the validated tessellate synthase gene of *A. mellea*. Structural domain analysis showed (Figure 5) that PKS 11290_t has a complete structural domain and shares a similar structural domain composition (SAT-KS-AT-PT-ACP-ACP-ACP-TE) as *H. erinaceus* HerA, *A. mellea* ArmB, and *A. cinnamomea* PKS63787. Phylogenetic analysis of PKSs (KS domain) from different species also indicated that 11290_t may be an orsellinic acid synthase, capable of synthesizing OA using acetyl-CoA and malonyl-CoA as substrates (Figure 5, Table S1).

### 3.4. Cytochrome P450 Monooxygenase (CYP) Family Analysis

Cytochrome P450s (CYPs) are enzymes that are ubiquitous in living organisms and play a role in important metabolic processes, such as development and biotic–nutrient interactions in most organisms [49]. Fungi possess a more diverse family of CYPs than plants, animals, or bacteria, with a wide range of substrates, strong catalytic ability, and a high frequency of participation, playing an important role in the biosynthesis of fungal natural products [50]. Various reactions such as hydroxylation, epoxidation, and sequential oxidation, catalyzed by P450, greatly enrich the chemical structures and biological activities of fungal natural products [51].

To further understand the functional gene composition of *I. lacteus*, the number and types of its P450 genes were analyzed. Based on domain and Pfam prediction, 106 P450 genes were identified in the *I. lacteus* genome (Table S2). The definitive classification of *I. lacteus* P450 was established by analyzing the evolutionary relationship between these 106 protein sequences and the representative basidiomycete P450 sequences in the fungal cytochrome P450 database. Family clustering analysis of the 106 CYPs revealed the presence of 19 CYP subfamilies and 1 indeterminate cluster (Figure 5). Among the identified CYP families, family CYP5037 had the highest number of members with 17 members, followed by the CYP5146 family (13), CYP5144 family (12), CYP5145 family (9), CYP5035 family (8), CYP620 family (7), CYP5152 family (6), CYP63 (5), CYP53 and CYP5058 families (4), CYP5142 and CYP5158 families (3), CYP5141, CYP5136, and CYP52 families (2), CYP539, CYP61, and CYP5148 families (1), along with 6 P450s dispersed in an indeterminate group, accounting for 5.83%. These unidentified P450s indicate the presence of potential new P450 types that require further analysis and characterization.

### 3.5. Genome-Wide Identification and Analysis of the MYB Gene Family

MYB transcription factors (MYB TFs) are a class of proteins encoded by genes, belonging to a family of transcription factor genes commonly found in eukaryotes [52]. The DNA-binding structural domains of MYB TFs are highly conserved and consist of 1–4 tandem incomplete repeat sequences, which are prone to the formation of helix-turn-horn-helix structures involved in the binding process of transcription factors to DNA [53]. According to the number of repetitive sequences contained, MYB TFs can be categorized into four subfamilies: 1R-MYB (R1/R2), 2R-MYB (R2R3), 3R-MYB (R1R2R3), and 4R-MYB (R1R2R3R1/R2) [54]. In plants, MYB TFs can regulate cell differentiation, cell morphology [55], and the cell cycle [56], and are also involved in regulating various physiological processes, such as secondary metabolic pathways [57]. MYB TFs in fungi are also widely involved in various physiological processes, including development and secondary metabolism regulation. For example, in *Aspergillus nidulans*, MYB TFs regulate asexual and sexual differentiation by promoting the production of conidia and asexual spores [58]. Sarikaya Bayram et al. found that MYB TFs regulate conidiation, promote alginate accumulation, and maintain cell wall integrity, which can effectively inhibit the asexual spores of *A. fumigatus* and prevent the occurrence of aspergillosis [59]. In addition, the MYB transcription factor gene families of important macrofungi, such as *C. sinensis* [60], *G. lucidum* [61], *Flammulina velutipes* [62], *A. camphorate* [63], *Wolfiporia cocos* [64], *P. ostreatus* [65], etc., have been characterized at the genome-wide level, and have been found to play important roles in the regulation of fungal development and metabolism. However, the identification of the MYB TFs gene family and biological functions of *I. lacteus* have not yet been reported.

**Figure 6:**
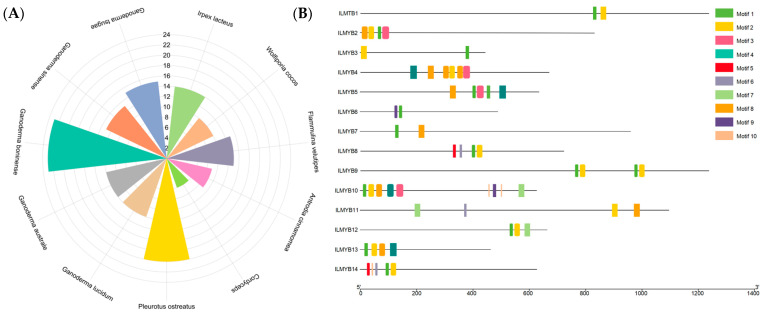
(**A**) Comparative plots of the number of MYB transcription factors in 11 fungal species; (**B**) prediction of motif, a MYB transcription factor of *I. lacteus*.

Based on the *I. lacteus* genomic data, 14 members of the MYB TFs gene family containing the MYB DNA-binding motif were screened by Pfam annotation and named ILMYB1 to ILMYB14. These genes encode amino acid sequences ranging in length from 441aa (ILMYB3) to 1240aa (ILMYB1). We compared their number with that of the MYB TFs of 10 other species of basidiomycetes and found that they corresponded to the number of MYB TFs possessed by basidiomycetes in general (Figure 6A). The results of the structural domain analysis (Figure 6B) showed that all MYB proteins in the ILMYB gene family share a common SANT conserved structural domain, and the conserved structural domains exist not only in the N-terminal region of the protein sequence but also in the intermediate sequence or C-terminal region of the protein. A total of 6 out of 14 MYB TFs are of the 1R type, 6 are of the 2R type, 1 is of the 3R type, and 1 is of the 4R type. ILMYB1, ILMYB3, ILMYB6, ILMYB8, ILMYB12, and ILMYB14 contain one SANT structural domain and belong to the 1R type of MYB transcription factors. ILMYB2, ILMYB7, ILMYB9, ILMYB10, ILMYB11, and ILMYB13 contain two SANT structural domains and belong to the 2R type of MYB transcription factors. ILMYB5 contains three SANT domains and belongs to the 3R type. ILMYB4 contains four SANT domains and belongs to the 4R type (Figure S7).

We constructed an evolutionary tree of 14 MYB TFs of *I. lacteus* together with functionally verified MYB proteins from *G. lucidum*, *P. ostreatus*, *C. sinensis*, and *A. fumigatus*, and the results showed that the 23 MYB TFs were divided into three branches (Figure S8). In branch II, ILMYB5 was clustered in the same branch as *P. ostreatus.* PoMYB15 and ILMYB1 were clustered with *C. sinensis* MYB-3. In branch III, ILMYB8 was clustered in the same branch as PoMYB12, and ILMYB10 was clustered in the same branch as PoMYB20. Therefore, we hypothesized that ILMYB8 and ILMYB10 of *I. lacteus* could enhance the response of *I. lacteus* mycelium to heat stress and thus promote the growth and development of the substrate.

**Figure 7:**
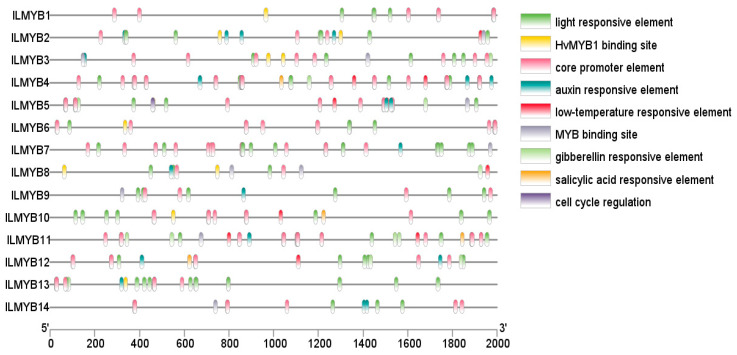
Screening identified nine cis-acting elements in the 2000 bp region upstream of the ILMYB transcription factor initiation codon (ATG) associated with secondary metabolism *I. lacteus*.

Further motif analysis of the MYB TFs revealed that ILMYB10 in the ILMYB gene family contained up to nine motifs, and the rest contained motifs ranging from two to six (Figure 6B). To analyze the potential biological functions of ILMYB genes, cis-acting elements in the 2000 bp region upstream of the ILMYB gene’s initiation codon (ATG) were identified (Figure 7). The screening yielded cis-acting elements related to photosensitive response and low-temperature stress response, as well as cis-acting elements related to phytohormone response and the growth and development of *I. lacteus*. The discovery of these cis-acting elements suggests that the MYB TF gene family plays an important role in the response to adverse environments, abiotic stress response, and growth and development of *I. lacteus*.

## 4. Discussion

In recent years, some researchers have begun to focus their attention on the study of *I. lacteus* secondary metabolites, using heterologous expression, chemical isolation, and other techniques to successfully isolate a variety of types of secondary metabolites, including terpenes, polyketides, furan derivatives, and a number of triterpene homologues, all of which exhibit good physiological activities. For example, Irpeksolactin- J, isolated from *I. lacteus*, showed selectivity and weak cytotoxicity against the human lung cancer cell line A549 and the human hepatocellular carcinoma cell line SMMC-7721 [66]. Irlactin I showed cytotoxicity against HL-60, SMMC-7721, A-549, MCF7, and SW480 cells [67]. Irpexoate B exhibited weak cytotoxicity against four human cancer cells (A-549, SMMC-7721, MCF-7, SW480) [68]. Inhibition of LPS-induced NO release from RAW 264.7 cells by irpexolaceus A, C, D, F, and G, irpexonjust B, and irpexlacte B [69].And these sesquiterpenoids and triterpenoids with multiple structures and multiple biological activities are their main secondary metabolites. Thus, we consider *I. lacteus* as basidiomycetes with great medicinal potential. Prior to our study, there were two reports on the genome sequences of *I. lacteus* strains. One reported a systematic study of the dye decolorizing peroxidase and manganese peroxidase gene families of *I. lacteus* strain F17 [70]. The other analyzed the molecular mechanism of lignocellulose pretreatment by *I. lacteus* CD2 and its implications based on genomic and transcriptomic analysis [71]. Both studies deepened our knowledge and understanding of lignocellulose degradation by *I. lacteus*. These findings help to further develop its potential for industrial and environmental applications. In this study, we found the *I. lacteus* Y1 genome to be 41.83Mb, which is slightly smaller compared to the previously sequenced *I. lacteus* F17 genome (44.36 Mb) and *I. lacteus* CD2 genome (43.16 Mb). However, the number of glycoside hydrolases (GHs) encoded by *I. lacteus* Y1 was higher, with 233 GHs, compared to 161 GHs in *I. lacteus* F17 and 182 GHs in *I. lacteus* CD2. This suggests that *I. lacteus* Y1, compared to *I. lacteus* F17 and *I. lacteus* CD2, may have a higher capacity for lignocellulose degradation. In this study, we identified and annotated important genes related to secondary metabolism. A total of 14 terpene synthase-related genes were identified, as well as 2 clusters of terpene biosynthetic genes with the potential to synthesize structurally complex products. We also identified four PKS genes, one of which may be a tesselate synthase capable of synthesizing tesselate. There are also eight NRPS-like enzymes in *I. lacteus* Y1. There are very few studies on NRPS in tamarins, so this area awaits further analysis and research.

The *I. lacteus* genome was screened for 106 P450 genes distributed among 19 CYP subfamilies and 1 indeterminate group. In recent years, several functional P450s have been identified in basidiomycetes. In *G. lucidum*, CYP5150L8 catalyzes the three-step biotransformation of lanosterol at the C-26 site for the synthesis of HLDOA [72]. CYP5139G1 oxidizes the C-28 of HLDOA to form 3,28-dihydroxy-oryzanol-8,24-dien-26-oic acid (DHLDOA). CYP512W2 can continue to use HLDOA as a substrate and oxidize it to form both type I and type II ganoderic acid [73]. Functional screening analysis showed that CYP5035 was involved in the fungal detoxification mechanism [74]. Thus, the different types of P450 we screened may play important regulatory roles in the biosynthesis of secondary metabolites and the growth and development of *I. lacteus*.

The MYB TFs family is evolutionarily conserved in plants, animals, and fungi, contributing to their growth and development [75]. We performed a genome-wide analysis of the MYB TFs family of *I. lacteus*, including structural domain prediction, phylogenetic relationships, conserved motif prediction, and cis-acting element prediction. This provides a functional annotation of the MYB TFs family of *I. lacteus* and serves as an informative reference for further functional validation.

## 5. Conclusions

In this study, we performed a genome-wide analysis of the important medicinal fungus *I. lacteus*. We performed de novo sequencing using a third-generation PacBio Sequel II sequencer and conducted a detailed functional annotation of the *I. lacteus* genome using several major databases. The chromosome-level assembly and functional annotation of the genome provide an important basis for studying the CAZymes of this mushroom, which will help in its artificial cultivation. The secondary metabolite biosynthesis genes in the *I. lacteus* genome contribute to our understanding of the complex secondary metabolite synthesis mechanism of *I. lacteus*. Cluster analysis reflected the diversity of *I. lacteus* P450 enzymes. The analysis of the MYB TFs gene family provided reference information for further functional validation of these genes. The present study not only enriches the genetic information of *I. lacteus*, but also provides important insights into the genome of mushrooms in the genus Irpex.

## Figures and Tables

**Table 1:** De novo genome assembly and features of *I. lacteus*.

Characteristics	Value
Total length (bp)	41,831,088
Contigs	55
Scaffolds	55
N50 (bp)	3,951,072
N90 (bp)	2,327,118
L50	5
L90	10
GC%	49.82

**Table 2:** Statistical table of the gene information.

Characteristics	Value
CDS number	13,135
CDS total length	20,105,286 bp
CDS density	0.314 genes per kb
CDS average length	1530 bp
Intergenetic region length	21,725,802 bp
CDS/Genome (coding percentage)	48.10%
Intergenetic length/Genome	51.90%
GC content in gene region	53.70%

**Table 3:** Putative BGCs responsible for secondary metabolites in the strain Y1.

Cluster No.	Location	Start (bp)	End (bp)	Core Gene ID	Core Gene Type
1	Chr2	1,966,880	1,968,009	1945_t	Terpene
2	Chr3	1,287,295	1,297,241	2437_t	PKS
3	Chr4	1,179,836	1,184,996	3714_t	NRPS-like
4	Chr5	1,262,257	1,266,817	5076_t	NRPS-like
5	Chr6	1,742,732	1,745,523	5909_t	Terpene
6	Chr6	2,091,664	2,095,641	6013_t	NRPS-like
7	Chr6	2,530,444	2,532,296	6158_t	Terpene
8	Chr6	2,907,033	2,908,352	6273_t	Terpene
9	Chr7	1,027,290	1,028,582	6816_t	Terpene
10	Chr7	1,562,469	1,566,947	7012_t	NRPS-like
11	Chr7	2,469,431	2,470,534	7340_t	Terpene
12	Chr7	2,471,371	2,472,669	7341_t	Terpene
13	Chr7	3,746,009	3,750,647	7760_t	PKS
14	Chr7	3,765,445	3,766,804	7766_t	PKS
15	Chr8	597,512	601,542	8028_t	NRPS-like
16	Chr8	705,376	709,477	8060_t	NRPS-like
17	Chr8	1,189,829	1,192,243	8219_t	Terpene
18	Chr8	2,059,227	2,060,877	8523_t	Terpene
19	Chr9	763,448	767,556	9069_t	NRPS-like
20	Chr10	637,671	639,055	10957_t	Terpene
21	Chr10	1,622,548	1,629,357	11290_t	PKS
22	Chr10	2,367,015	2,368,086	11546_t	Terpene
23	Chr10	2,369,863	2,371,065	11547_t	Terpene
24	Chr10	2,422,443	2,423,554	11558_t	Terpene
25	Chr10	2,425,282	2,427,793	11559_t	Terpene
26	Chr11	2,261,230	2,265,279	12363_t	NRPS-like

## Data Availability

The *I. lacteus* genomic data have been deposited under accession JBIDZI000000000 in GenBank. The genome raw sequencing data and the reported assembly are associated with NCBI BioProject: PRJNA1168250 and BioSample: SAMN44032060 within GenBank.

## Supplementary Materials

The following supporting information can be downloaded at: https://www.mdpi.com/article/10.3390/jof10120846/s1, Table S1: the analyzed PKSs in phylogenetic tree; Table S2: identification of cytochrome P450 genes in *I. lacteus* genome; Figure S1: ITS alignment of the strain Y1; Figure S2: GC content and coverage correlation analysis; Figure S3: number of annotated genes in 20 species; Figure S4: Cluster of Orthologous Groups of proteins (COG); Figure S5: statistical map of functional annotation classification based on GO database; Figure S6: Kyoto Encyclopedia of Genes and Genomes (KEGG); Figure S7: prediction of the structural domains of the MYB transcription factor in *I. lacteus*; Figure S8: phylogenetic tree of MYB proteins in *I. lacteus*.
