# Supplementary material for: Genome Sequencing and Metabolic Potential Analysis of Irpex lacteus
Source: J Fungi (Basel). 2024 Dec 7;10(12):846. doi: 10.3390/jof10120846 (PMC11677564; doi:10.3390/jof10120846)
Supplement: Supplementary file 1 [file jof-10-00846-s001.zip › jof-3268430-supplementary.pdf]

Genome Sequencing and Metabolic Potential Analysis of *Irpex lacteus*

Yue,Wang [1], Yingce Duan [1], Menghan Zhang [1], Chaoqin Liang [2], Wenli Li [3] , Chengwei Liu [1,*] and Ying Ye [2*]

1 Key Laboratory for Enzyme and Enzyme-like Material Engineering of Heilongjiang, College of Life Science, Northeast Forestry University, Harbin 150040, China
2 Hubei Key Laboratory of Natural Medicinal Chemistry and Resource Evaluation, School of Pharmacy, Tongji Medical College, Huazhong University of Science and Technology, Wuhan 430030, China
3 State Key Laboratory for Crop Stress Resistance and High Efficiency Production, Shaanxi Key Laboratory of Natural Products & Chemical Biology, College of Chemistry & Pharmacy, Northwest A&F University, Yangling 712100, China
* Correspondence: liuchw@nefu.edu.cn (C.L.), ying_ye@hust.edu.cn (Y.Y.)

Table S1. The analyzed PKSs in phylogenetic tree.

Organism	Enzyme	Accession number	Proposed domain structure
Antrodia cinnamomea	PKS63787	AST08390.1	SAT KS AT PT ACP ACP TE
Armillaria mellea	ArmB	I3ZNU9.1	SAT KS AT PT ACP ACP TE
Aspergillus fumigatus	Alb1	AAC39471.1	SAT KS AT PT ACP ACP TE
Aspergillus fumigatus	FccA	Q4WA61.1	SAT KS AT DH ACP
Aspergillus nidulans	WA	CAA46695.2	SAT KS AT PT ACP ACP TE
Aspergillus nidulans	MdpG	Q5BH30.1	SAT KS AT DH ACP
Aspergillus nidulans	AptA	XP_663604.1	SAT KS AT PT ACP
Aspergillus niger	AdaA	G3KLH6.1	SAT KS AT DH ACP
Aspergillus nidulans	PkfA	Q5B8A0.1	SAT KS AT DH ACP
Aspergillus niger	AlbA	A2QUI2.1	SAT KS AT DH ACP ACP TE
Aspergillus parasiticus	AflC	Q12053.1	SAT KS AT PT ACP TE
Aspergillus terreus	ACAS	XP_001217072.1	SAT KS AT PT ACP
Coprinopsis cinerea	CC1G	XP_001835415.2	KS AT PT ACP TE
Cortinarius odorifer	CoPKS1	P9WES1	KS AT DH ACP AB
Cortinarius odorifer	CoPKS4	P9WES2	KS AT DH ACP AB
Penicillium aethiopicum	GsfA	D7PI15.1	SAT KS AT DH ACP
Penicillium brevicompactum	MpaC	F1DBA9.1	ACP KS AT DH ACP AB
BY1	PKS1	APH07629.1	SAT KS AT DH ACP TE
BY1	PKS2	APH07628.1	SAT KS AT DH ACP ACPACP TE
Aspergillus oryzae	PKSA	BAE71314.1	SAT KS AT PT ACP TE
Agaricus bisporus var. bisporus H97 v2.0	PKS1	202648	SAT KS AT PT ACP ACP ACP
Auricularia delicata SS-5	PKS1	167360	KS AT DH KR ACP RED
Fomitiporia mediterranea	PKS1	144436	X KS AT PT ACP TE
Ganoderma sp.10597	PKS1	86609	KS AT PT ACP TE
Fomitopsis pinicola SS1	PKS1	83434	SAT KS AT PT ACP TE
Laccaria bicolor	PKS1	XP_001876029.1	SAT KS AT PT ACP TE
Schizophyllum commune	PKS1	XP_003038401.1	SAT KS AT PT ACP TE
Ustilago maydis	PKS3	XP_011390610.1	KS X PT ACP ACP TE
Laetiporus sulphureus	LpaA	QNJ99675.1	KS AT DH MT ER KR ACP TE
Trametes versicolor	PKS1	XP_008041566.1	KS AT DH ACP ACP TE
Trametes chinabarina	PKS1	CDO73915.1	KS AT PT ACP ACP TE
Stereum hirsutum	PKS1	XP_007307184.1	SAT KS AT DH ACP ACP ACP TE
Fibroporia radiculosa	PKS1	XP_012179384.1	SAT KS AT PT ACP ACP TE
Amanita muscaria	PKS1	KIL57002.1	SAT KS AT PT ACP ACP
Grifola frondosa	PKS1	BAO20284.1	SAT KS AT DH ACP ACP TE

Table S2. Identification of cytochrome P450 genes in *I. lacteus* genome.

Query	Hit type	PSSM-ID	From	To	E-Value	Bitscore	Accession	Short name
79_t	specific	410688	64	490	0	543.324	cd11065	CYP64-like
83_t	specific	410688	80	489	3.17E-176	502.108	cd11065	CYP64-like
854_t	specific	410693	148	551	9.52E-106	325.053	cd11070	CYP56-like
1016_t	specific	410688	81	496	1.30E-170	488.241	cd11065	CYP64-like
1017_t	specific	410688	79	491	2.47E-155	449.335	cd11065	CYP64-like
1126_t	specific	410688	65	512	4.08E-176	502.878	cd11065	CYP64-like
1144_t	specific	410688	1	391	1.73E-146	422.757	cd11065	CYP64-like
1145_t	specific	410688	85	515	5.73E-156	451.647	cd11065	CYP64-like
1146_t	specific	410688	80	508	6.15E-168	481.692	cd11065	CYP64-like
1609_t	specific	410667	64	501	4.02E-175	499.897	cd11041	CYP503A1-like
1612_t	specific	410667	64	503	5.15E-162	466.385	cd11041	CYP503A1-like
1613_t	specific	410667	64	501	2.35E-164	472.548	cd11041	CYP503A1-like
1657_t	specific	410685	486	862	3.39E-128	392.002	cd11062	CYP58-like
1658_t	specific	410685	7	376	3.64E-133	387.765	cd11062	CYP58-like
1684_t	specific	410685	77	489	2.60E-151	438.611	cd11062	CYP58-like
1852_t	superfamily	372343	206	370	1.89E-16	76.2663	cl24253	CYP67-like
1934_t	specific	410688	79	503	4.58E-167	479.381	cd11065	CYP64-like
1958_t	specific	410688	66	478	5.64E-165	473.218	cd11065	CYP64-like
1961_t	specific	410688	74	501	3.70E-168	482.463	cd11065	CYP64-like
1962_t	specific	410692	66	530	5.72E-143	419.367	cd11069	CYP_FUM15-like
2517_t	specific	410692	84	536	2.03E-168	484.851	cd11069	CYP_FUM15-like
2524_t	specific	410692	83	535	1.08E-166	480.229	cd11069	CYP_FUM15-like
2525_t	specific	410692	71	522	1.24E-169	487.162	cd11069	CYP_FUM15-like
2554_t	specific	410688	76	504	2.11E-141	414.282	cd11065	CYP64-like
2739_t	specific	410688	65	482	2.52E-154	445.483	cd11065	CYP64-like
2839_t	specific	410692	65	527	3.28E-143	420.138	cd11069	CYP_FUM15-like
2846_t	specific	410688	1229	1657	2.82E-173	528.301	cd11065	CYP64-like
2966_t	specific	410692	71	531	5.95E-140	411.663	cd11069	CYP_FUM15-like
3822_t	specific	410688	62	485	1.02E-170	487.855	cd11065	CYP64-like
3896_t	specific	410688	65	481	3.42E-164	473.603	cd11065	CYP64-like
4217_t	specific	410692	65	525	2.33E-150	438.242	cd11069	CYP_FUM15-like
4226_t	specific	410688	68	488	6.27E-161	481.307	cd11065	CYP64-like
4227_t	specific	410688	79	506	1.66E-155	450.106	cd11065	CYP64-like
4236_t	specific	410688	1	420	1.48E-165	472.447	cd11065	CYP64-like
4237_t	specific	410688	69	498	0	522.908	cd11065	CYP64-like
4238_t	specific	410688	70	497	9.13E-155	447.795	cd11065	CYP64-like

4240_t	specific	410688	64	491	2.79E-162	467.055	cd11065	CYP64-like
4371_t	specific	410688	64	482	8.51E-150	435.083	cd11065	CYP64-like
4372_t	specific	410688	62	486	9.80E-159	457.425	cd11065	CYP64-like
4497_t	specific	410688	69	498	1.43E-173	495.945	cd11065	CYP64-like
4523_t	specific	410688	68	523	2.60E-176	503.648	cd11065	CYP64-like
4524_t	specific	410688	69	493	1.13E-167	480.537	cd11065	CYP64-like
4708_t	specific	410688	84	569	4.57E-147	430.075	cd11065	CYP64-like
5308_t	specific	410686	130	601	1.76E-166	480.902	cd11063	CYP52
5541_t	specific	410688	79	498	1.28E-162	468.21	cd11065	CYP64-like
6102_t	specific	410684	85	546	2.64E-141	415.083	cd11061	CYP67-like
6139_t	specific	410684	85	545	6.81E-133	393.897	cd11061	CYP67-like
6224_t	specific	410691	25	451	0	570.666	cd11068	CYP120A1
6561_t	specific	410686	126	592	3.10E-175	507.866	cd11063	CYP52
6562_t	specific	410686	129	595	4.91E-173	497.465	cd11063	CYP52
6566_t	specific	410686	129	595	1.52E-176	506.325	cd11063	CYP52
6629_t	specific	410693	82	497	3.24E-107	326.979	cd11070	CYP56-like
6840_t	specific	410688	1	389	2.39E-150	432.387	cd11065	CYP64-like
7003_t	specific	410691	25	459	0	561.806	cd11068	CYP120A1
7013_t	specific	410691	25	452	0	663.499	cd11068	CYP120A1
7021_t	specific	410668	83	539	0	545.659	cd11042	CYP51-like
7177_t	specific	410692	73	547	1.51E-149	437.086	cd11069	CYP_FUM15-like
7955_t	specific	410688	99	530	3.35E-162	468.21	cd11065	CYP64-like
7972_t	specific	410688	80	507	3.47E-166	477.455	cd11065	CYP64-like
7974_t	specific	410688	29	376	3.50E-136	395.407	cd11065	CYP64-like
7988_t	specific	410688	76	490	1.58E-157	454.343	cd11065	CYP64-like
7993_t	specific	410688	86	533	9.55E-173	495.174	cd11065	CYP64-like
7994_t	specific	410688	98	525	7.63E-156	451.647	cd11065	CYP64-like
7995_t	specific	410688	101	529	0	544.094	cd11065	CYP64-like
8000_t	specific	410688	1	388	5.44E-151	434.313	cd11065	CYP64-like
8001_t	specific	410688	98	525	5.53E-180	513.278	cd11065	CYP64-like
8036_t	specific	410688	62	487	2.34E-166	478.225	cd11065	CYP64-like
8037_t	specific	410688	115	540	2.79E-165	477.84	cd11065	CYP64-like
8039_t	specific	410688	62	487	1.09E-171	492.093	cd11065	CYP64-like
8394_t	specific	410688	62	504	6.96E-173	494.404	cd11065	CYP64-like
8486_t	specific	410688	1	416	2.18E-149	431.231	cd11065	CYP64-like
8741_t	specific	410684	133	569	1.03E-128	383.497	cd11061	CYP67-like
8742_t	specific	410684	131	546	2.35E-126	376.563	cd11061	CYP67-like
8746_t	specific	410667	57	513	2.03E-163	470.622	cd11041	CYP503A1-like
8832_t	specific	410684	129	550	5.82E-131	388.119	cd11061	CYP67-like
8843_t	specific	410684	130	570	3.11E-127	379.645	cd11061	CYP67-like
8844_t	specific	410684	130	549	2.10E-133	394.668	cd11061	CYP67-like
8846_t	specific	410684	80	565	5.93E-134	396.979	cd11061	CYP67-like
8847_t	specific	410684	80	549	1.63E-139	410.461	cd11061	CYP67-like
9122_t	specific	410686	116	580	1.46E-169	488.22	cd11063	CYP52
9268_t	specific	410688	59	480	6.95E-142	414.282	cd11065	CYP64-like
9362_t	specific	410703	92	501	0	591.143	cd11082	CYP61_CYP710
9807_t	specific	410667	60	482	3.34E-154	445.969	cd11041	CYP503A1-like

9858_t	superfa mily	475119	674	768	0.0002367 34	43.7159	cd11065	CYP64-like
10076_t	specific	410688	73	500	0	563.354	cd11065	CYP64-like
10895_t	specific	410688	81	504	1.29E-176	503.648	cd11065	CYP64-like
10931_t	specific	410691	36	455	0	618.815	cd11068	CYP120A1
10956_t	specific	410688	62	483	0	535.235	cd11065	CYP64-like
10958_t	specific	410688	63	487	4.87E-167	480.151	cd11065	CYP64-like
11332_t	specific	410688	70	495	2.25E-178	507.886	cd11065	CYP64-like
11450_t	specific	410688	60	503	1.69E-172	493.248	cd11065	CYP64-like
11811_t	specific	410684	133	553	8.60E-130	385.808	cd11061	CYP67-like
11892_t	specific	410684	126	556	2.41E-127	379.26	cd11061	CYP67-like
12184_t	specific	410684	124	518	2.67E-115	365.778	cd11061	CYP67-like
12258_t	specific	410688	69	508	1.62E-161	464.743	cd11065	CYP64-like
12443_t	specific	410688	62	485	9.75E-165	472.833	cd11065	CYP64-like
12448_t	specific	410688	63	475	1.17E-157	454.343	cd11065	CYP64-like
12478_t	specific	410688	62	486	1.17E-164	472.447	cd11065	CYP64-like
12480_t	specific	410688	140	565	1.58E-162	470.136	cd11065	CYP64-like
12481_t	specific	410688	73	496	4.17E-162	466.669	cd11065	CYP64-like
12482_t	specific	410688	62	466	1.25E-154	446.254	cd11065	CYP64-like
12487_t	specific	410688	63	487	6.55E-154	445.483	cd11065	CYP64-like
12501_t	specific	410691	35	430	0	548.709	cd11068	CYP120A1
12502_t	specific	410691	26	459	0	598.4	cd11068	CYP120A1
12503_t	specific	410691	25	455	0	621.127	cd11068	CYP120A1
12589_t	specific	410688	68	497	4.59E-177	504.804	cd11065	CYP64-like

Descriptions | Graphic Summary | Alignments | Taxonomy

Sequences producing significant alignments Download Select columns Show 100

select all *100 sequences selected* GenBank Graphics Distance tree of results MSA Viewer

Description	Scientific Name	Max Score	Total Score	Query Cover	E value	Per. Ident	Acc. Len	Accession
Uncultured Irpex clone GM99 internal transcribed spacer 1, partial sequence; 5.8S ribosomal RNA gene and intern...	uncultured Irpex	1177	1177	100%	0.0	99.69%	649	MG462835.1
Irpex lacteus voucher CLZhao 9878 internal transcribed spacer 1, partial sequence; 5.8S ribosomal RNA gene and ...	Irpex lacteus	1177	1177	99%	0.0	99.84%	682	MT177300.1
Irpex lacteus voucher CLZhao 9398 internal transcribed spacer 1, partial sequence; 5.8S ribosomal RNA gene and ...	Irpex lacteus	1177	1177	99%	0.0	99.84%	679	MT177293.1
Irpex lacteus strain WZ-149 small subunit ribosomal RNA gene, partial sequence; internal transcribed spacer 1, 5.8...	Irpex lacteus	1173	1173	99%	0.0	99.69%	679	MN856293.1
Fungal sp. isolate 31 internal transcribed spacer 1, partial sequence; 5.8S ribosomal RNA gene and internal transcr...	fungal sp.	1171	1171	99%	0.0	99.84%	651	PQ577768.1
Irpex lacteus voucher CLZhao 9922 internal transcribed spacer 1, partial sequence; 5.8S ribosomal RNA gene and ...	Irpex lacteus	1171	1171	100%	0.0	99.53%	673	MT177303.1
Irpex lacteus voucher CLZhao 81 18S ribosomal RNA gene, internal transcribed spacer 1, 5.8S ribosomal RNA gen...	Irpex lacteus	1171	1171	99%	0.0	99.69%	675	MG231699.1
Irpex lacteus voucher CLZhao 11017 small subunit ribosomal RNA gene, partial sequence; internal transcribed spa...	Irpex lacteus	1171	1171	100%	0.0	99.53%	671	MW742571.[illegible]
Irpex lacteus voucher CLZhao 9888 internal transcribed spacer 1, partial sequence; 5.8S ribosomal RNA gene and ...	Irpex lacteus	1171	1171	99%	0.0	99.69%	679	MT177302.1
Irpex lacteus voucher CLZhao 11953 internal transcribed spacer 1, partial sequence; 5.8S ribosomal RNA gene an...	Irpex lacteus	1171	1171	99%	0.0	99.69%	672	MW578308.[illegible]
Irpex lacteus strain WZ-166 small subunit ribosomal RNA gene, partial sequence; internal transcribed spacer 1, 5.8...	Irpex lacteus	1170	1170	100%	0.0	99.53%	683	MN856303.1
Irpex lacteus strain YF3 internal transcribed spacer 1, partial sequence; 5.8S ribosomal RNA gene and internal tran...	Irpex lacteus	1170	1170	99%	0.0	99.69%	661	OQ933276.1
Irpex lacteus strain FWJ01 internal transcribed spacer 1, partial sequence; 5.8S ribosomal RNA gene and internal t...	Irpex lacteus	1168	1168	100%	0.0	99.38%	711	OK184563.1
Irpex lacteus voucher CLZhao 917 18S ribosomal RNA gene, internal transcribed spacer 1, 5.8S ribosomal RNA ge...	Irpex lacteus	1168	1168	99%	0.0	99.53%	666	MG231704.1
Irpex lacteus voucher CLZhao 922 18S ribosomal RNA gene, internal transcribed spacer 1, 5.8S ribosomal RNA ge...	Irpex lacteus	1168	1168	100%	0.0	99.38%	668	MG231705.1
Irpex sp. E9126b internal transcribed spacer 1, partial sequence; 5.8S ribosomal RNA gene and internal transcribe...	Irpex sp. E9126b	1168	1168	99%	0.0	99.53%	661	JN615247.1

Figure S1. ITS alignment of the strain Y1.The ITS of strain Y1 was aligned to the nr database of NCBI.

The ITS of strain Y1
CGAGTTTTGACGGGTTGTAGCTGGCCTCTCACGAGGCATGTGCACGCCTGGCTCA
TCCACTCTTAACCTCTGTGCACTTTATGTAAGAGAAAAAAATGGTGGAAGCTTCC
AGGATCTCGCGAGAGGTCTTCGGTTGAACAAGCCGTTTTTCTTTCTTATGTTTTACT
ACAAACGCTTCAGTTATAGAATGTCAACTGTGTATAACACATTTATATACAACTTT
CAGCAACGGATCTCTTGGCTCTCGCATCGATGAAGAACGCAGCGAAATGCGATA
AGTAATGTGAATTGCAGAATTCAGTGAATCATCGAATCTTTGAACGCACCTTGCA
CTCCTTGGTATTCCGAGGAGTATGCCTGTTTGAGTCTCATGGTATTCTCAACCCCT
AAATTTTTGTAATGAAGGTTTAGCGGGCTTGGACTTGGAGGTTGTGTCGGCCCTTG
TCGGTCGACTCCTCTGAAATGCATTAGCGTGAATCTTACGGATCGCCTTCAGTGTG
ATAATTATCTGCGCTGTGGTGTTGAAGTATTTATGGTGTTCATGCTTCGAACCGTC
TCCTTGCCGAGACAATCATTTGACAATCTGAGCTCAAATCAGGTAGGACTACCCG
CTGAACTTAAGCATATCAAAAGGCGGAGGGAA

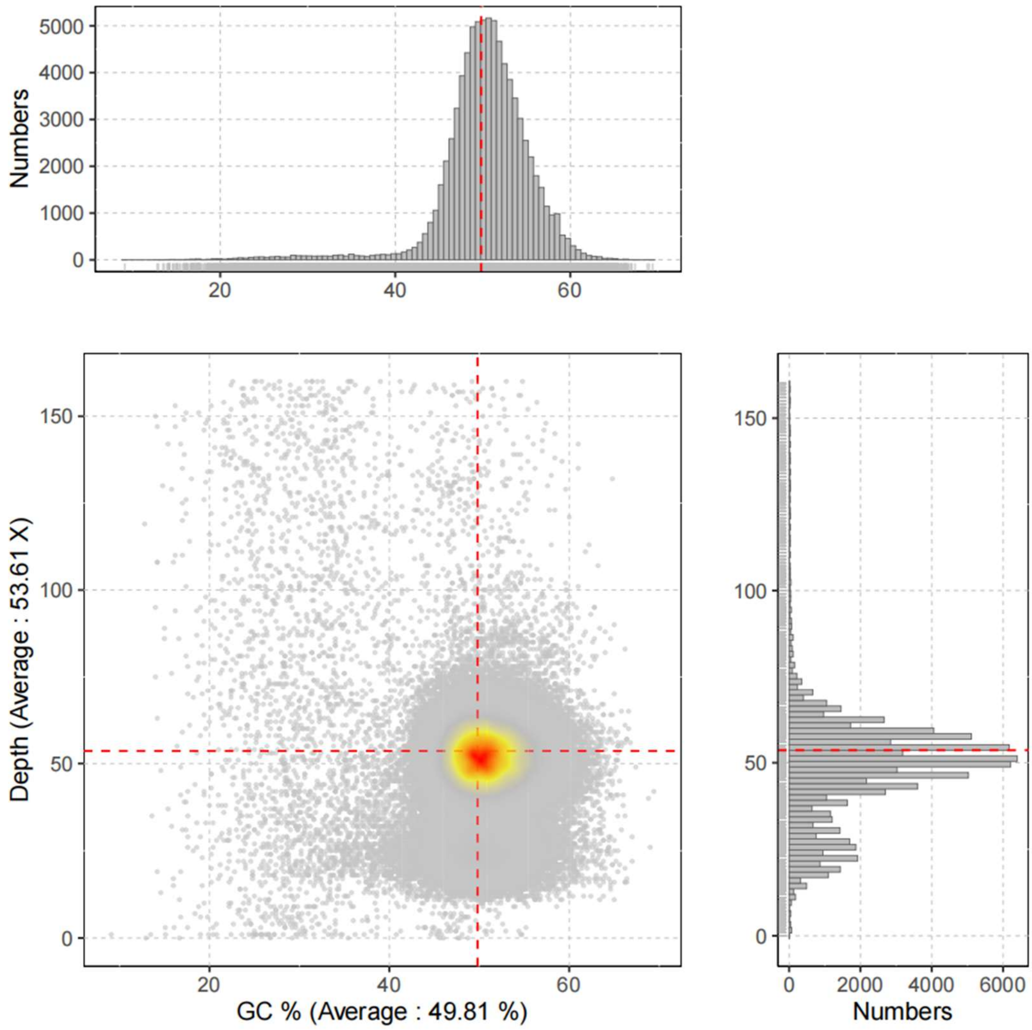

Figure S2. GC content and coverage correlation analysis.

The horizontal coordinate is the GC content and the vertical coordinate is the mean depth. The scatterplot exhibits a shape approximating a Poisson distribution, indicating that the GC bias during sequencing was not severe.

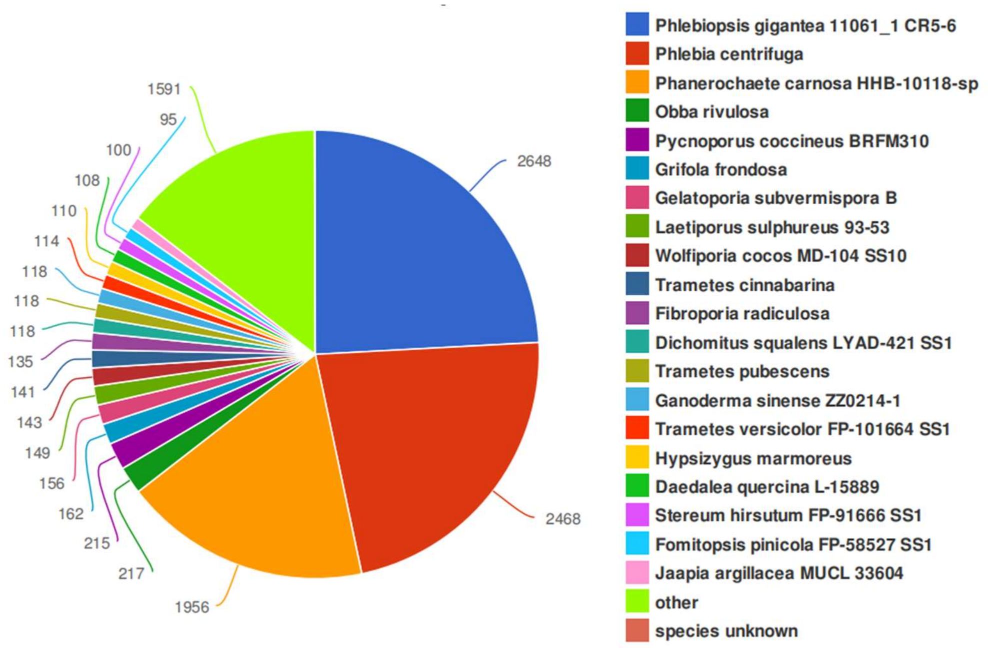

Figure S3. Species distribution map of Nr database alignment to sequences.

According to the results of the Nr library match, the top 10 species were counted and the rest were classified as other species, and the distribution of these species was mapped according to their proportion.

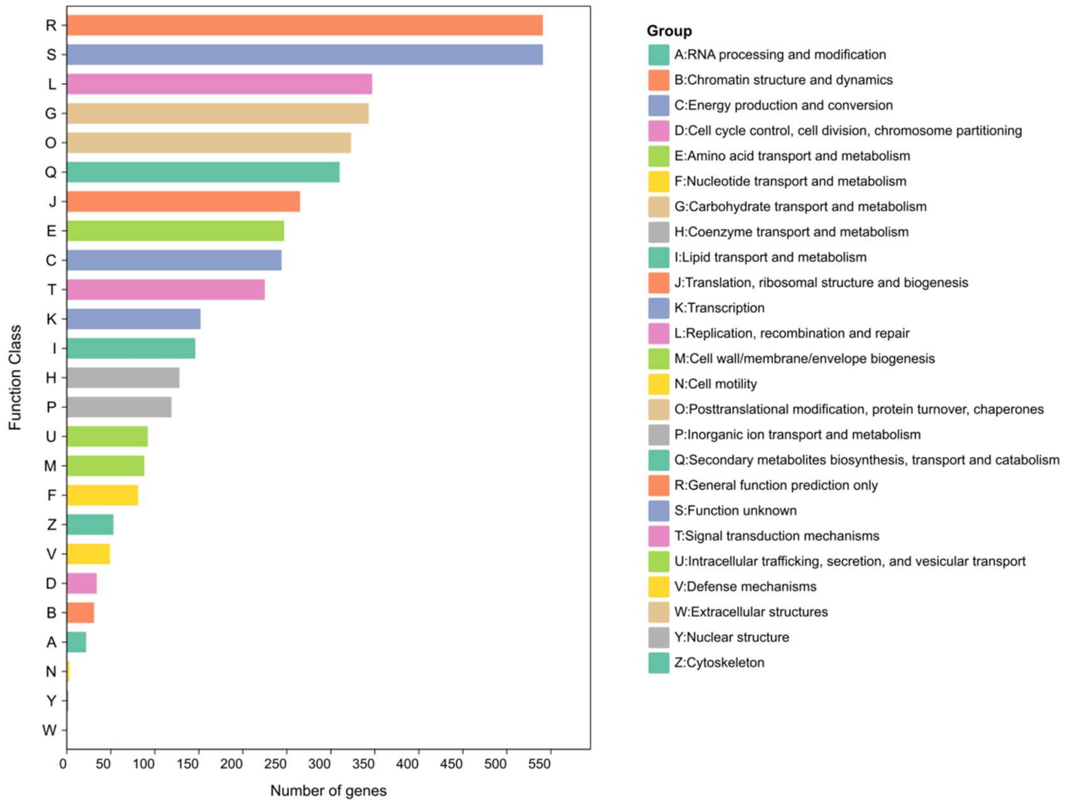

Figure S4. Cluster of Orthologous Groups of proteins (COG).

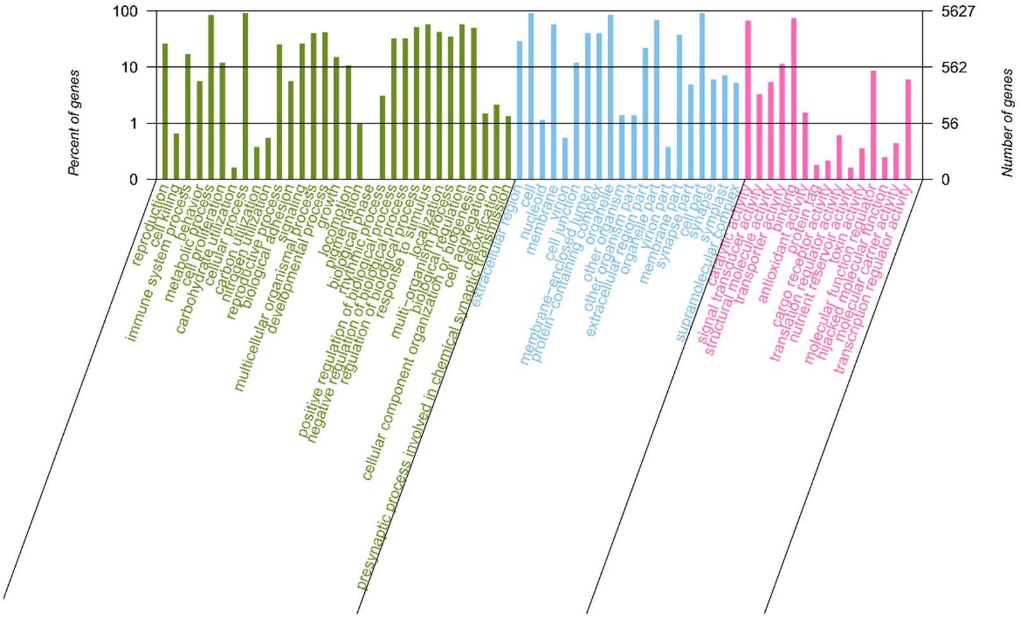

Figure S5. Statistical map of functional annotation classification based on GO database.

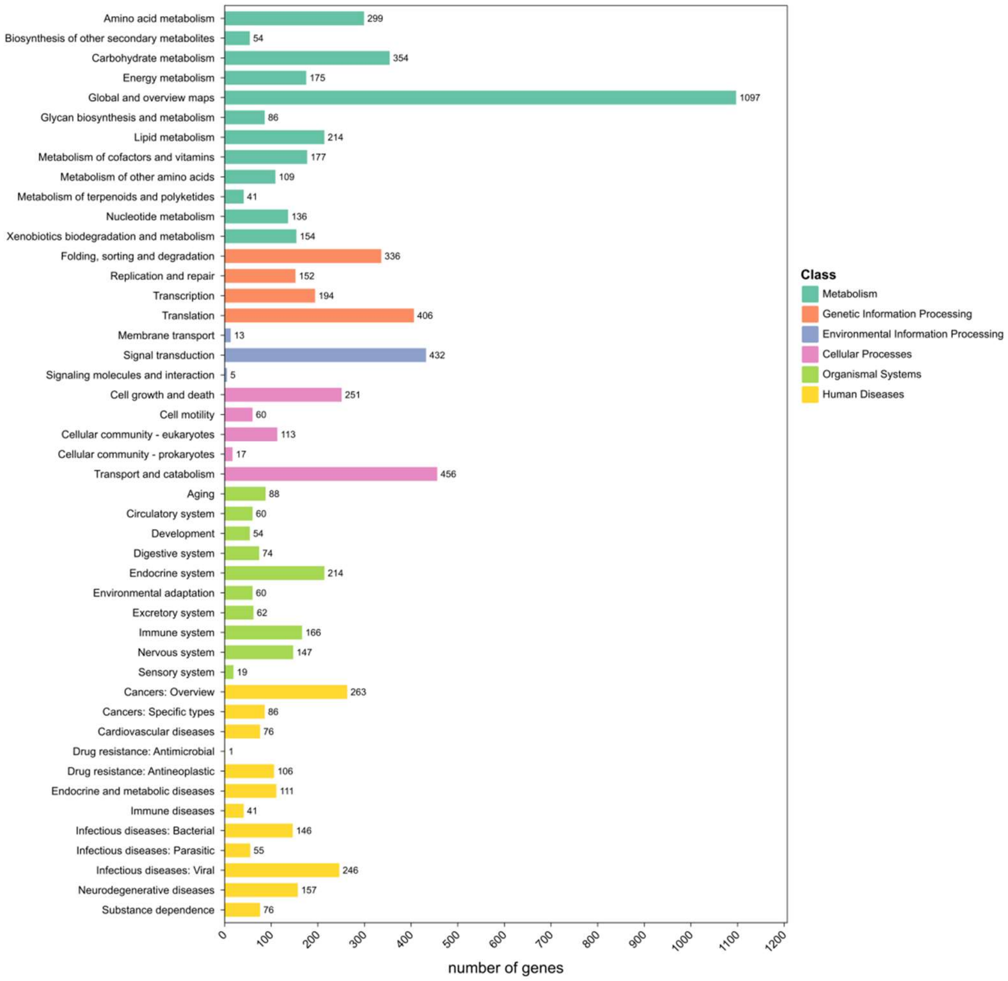

Figure S6. Kyoto Encyclopedia of Genes and Genomes (KEGG).

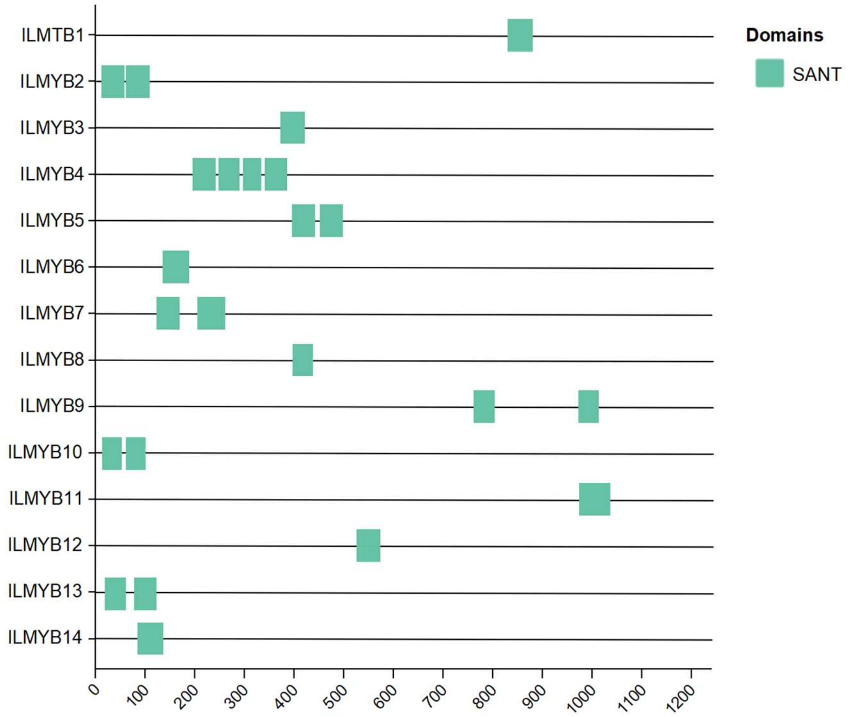

Figure S7. Schematic diagram of the predicted structural domains of the MYB transcription factor SANT in *I. lacteus*. The number of squares represents the number of structural domains possessed.

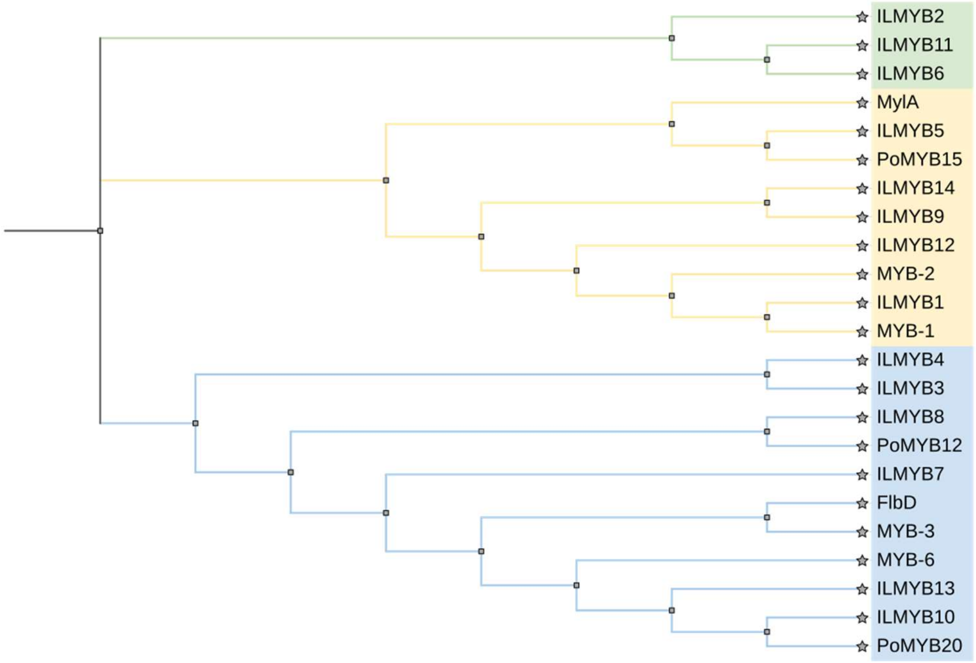

Figure S8. Phylogenetic tree of MYB proteins with known functions in *I. lacteus* and some fungi.
